# The Preclinical Pharmacological Study of a Novel Long-Acting Local Anesthetic, a Fixed-Dose Combination of QX-OH/Levobupivacaine, in Rats

**DOI:** 10.3389/fphar.2019.00895

**Published:** 2019-08-15

**Authors:** YuJun Zhang, QinQin Yin, DeYing Gong, Yi Kang, Jun Yang, Jin Liu, WenSheng Zhang

**Affiliations:** ^1^Laboratory of Anesthesia and Critical Care Medicine, Department of Anesthesiology, Translational Neuroscience Center, West China Hospital, Sichuan University, Chengdu, China; ^2^Sichuan Engineering Laboratory of Transformation Medicine of Anesthesiology, West China Hospital, Sichuan University, Chengdu, China

**Keywords:** preclinical drug development, preclinical pharmacokinetics, long-acting local anesthetic, fixed-dose combination, QX-OH

## Abstract

**Introduction:** Previous studies demonstrated that 35 mM QX-OH/10 mM Levobupivacaine (LL-1), a fixed-dose combination, produced a long-acting effect in rat local anesthesia models. All preclinical pharmacodynamic results indicated that LL-1 had potential for postsurgical pain treatment. The objective of this study was to investigate the pharmacokinetics of LL-1. Then, the possible mechanism of the extended duration by the combination was examined.

**Methods and Results:** All experiments were examined and approved by the Committee of Animal Care of the West China Hospital Sichuan University (Ethical approval number, 2015014A). The compound action potentials were recorded to verify the pharmacodynamic result in ex vivo. In frog sciatic nerve, LL-1 produced an effective inhibition with rapid onset time. The concentration-time profiles of LL-1 were determined in plasma and local tissues after sciatic nerve block. The maximum concentration of QX-OH and levobupivacaine were 727.22 ± 43.38 µg/g and 256.02 ± 28.52 µg/g in muscle, 634.26 ± 36.04 µg/g and 429.63 ± 48.64 µg/g in sciatic nerve, and 711.71 ± 25.14 ng/ml and 114.40 ± 10.19 ng/ml in plasma, respectively. The absorption of QX-OH into circulation was very rapid at 0.71 ± 0.06 h, which was faster than that of levobupivacaine (4.11 ± 0.39 h, p = 0.003). The half-time of QX-OH in plasma and local tissues had no significant difference (p = 0.329), with the values of 2.64 h, 3.20 h, and 3.79 h in plasma, muscle, and sciatic nerve, respectively. The elimination profile of levobupivacaine differed from that of QX-OH, which was slower eliminated from plasma (4.89 ± 1.77 h, p = 0.036) than from muscle (1.38 ± 0.60 h) or sciatic nerve (1.28 ± 0.74 h). When levobupivacaine was used alone, the Tmax in plasma was 1.07 ± 0.16 h. Interestingly, the Tmax of levobupivacaine in the plasma was increased by four times in combination with QX-OH (4.11 ± 0.39 h). Levobupivacaine promotes cellular QX-OH uptake.

**Conclusion:** The preclinical pharmacokinetic study of LL-1 in the rat plasma, muscle, and sciatic nerve was accomplished. Then, the possible mechanism of the prolonged duration was that QX-OH delayed the absorption of levobupivacaine from the injection site into circulation, and levobupivacaine accelerated QX-OH to accumulate into cells.

## Introduction

More than 80% of patients experience an acute pain after surgery, and in 75% of them, the pain severity is either moderate or extreme (Chou et al., 2016). The annual volume of surgeries has reached more than 300 million worldwide, suggesting that there are at least 200 million patients who will suffer an acute postsurgical pain every year (Weiser et al., 2016). Poor postsurgical pain management can cause a multitude of complications, including cardiopulmonary complications, prolonged hospital stays, development of chronic pain, and drug addiction (Kehlet et al., 2006). Multimodal analgesia is a primary pain control method in clinics that combines different analgesic drugs and techniques to generate a synergistic effect by utilizing distinct mechanisms of action and affecting different pain pathways (Kehlet and Dahl, 1993; Chou et al., 2016). Local anesthetics that provide adequate pain control with limited side effects are increasingly used in clinical practice (Wick et al., 2017). However, the short duration of local anesthetics typically requires continuous catheter infusion, which can be associated with complications, such as infection, dislodgement, leakage or nerve injury (Bomberg et al., 2016; Ilfeld, 2017). Thus, to develop a long-acting local anesthetic for postsurgical pain management is an unmet medical need (Santamaria et al., 2017).

In recent years, efforts to develop a novel long-acting local anesthetic included research on new drug molecules (Padera et al., 2006; Lim et al., 2007; Ries et al., 2009; Rodriguez-Navarro et al., 2011; Banerjee et al., 2015; Zhang et al., 2017b), adjuvant drugs (Kito et al., 1998; Sakamoto et al., 2016; Zhang et al., 2018), and sustained-release formulations (Ohri et al., 2013; Ramos Campos et al., 2013; Burbridge and Jaffe, 2015; Zhao et al., 2017b). In previous studies, we showed that QX-OH (Invention Patent, China, ZL201410688865.1), a quaternary lidocaine derivative, maintained the local anesthetic effect over a long period in rats (Zhang et al., 2017b). Furthermore, we found that the duration of QX-OH can be extended in combination with levobupivacaine (Levo-Bupi), and the longest duration with moderate local toxicity was achieved at the constant concentration ratio of 35 mM/10 mM (QX-OH/Levo-Bupi, denoted as LL-1) (Zhao et al., 2017a; Yin et al., 2019). In our previous work (Zhao et al., 2017a), LL-1 had a longer duration than Exparel™, a sustained-release formulation of bupivacaine, in rat models for sciatic nerve block and total knee surgery. These preclinical pharmacodynamic data indicated the potential of LL-1 as an effective treatment for postsurgical pain.

The objective of this study was to assess the pharmacokinetics of LL-1 in rats. We further investigated the underlying mechanism of the extended duration of the local anesthetic effect achieved by the fixed dose combination.

## Materials and Methods

### Materials

QX-OH (purity: 99.8%) was synthesized according to the protocol described in our previous study (Zhang et al., 2017b). QX-314, lidocaine, levobupivacaine, acetonitrile (HPLC grade), and formic acid (HPLC grade) were purchased from Sigma-Aldrich Co., Ltd. (MO, USA). Ropivacaine was purchased from the China National Institutes for Food and Drug Control. Isoflurane was bought from Hebei Yipin Pharmaceutical Co., Ltd. (Hebei, China). DMEM with high glucose, penicillin–streptomycin, and phosphate buffered saline (PBS) were purchased from Thermo Fisher Scientific Inc. Ringer solution was bought from Procell Life Science & Technology Co., Ltd. (Wuhan, China). The LL-1 was prepared by diluting the mixture of QX-OH powder and Levo-Bupi powder at ratio of 35 mM/10 mM in ultrapure water. Ultrapure water was prepared by Milli-Q^®^ water purification system (Merck Millipore, Darmstadt, Germany).

### Animals

The weight of young adult Sprague-Dawley rats at range of 270 to 330 g and frogs were purchased from Dossy Biological Technology Co., Ltd. (Chengdu, China). Rats were maintained in 5 per cage with separate ventilation, uncontrolled food and water supply, controlled temperature and humidity under 12-h light/dark cycle (lights on at 7:00 a.m.). The total number of animals used in the study was 160 for rat and 9 for frog. In each group, the blood samples were collected from 10 rats (half male and half female), respectively. To determine drug concentration in local tissues, 110 male rats were randomized into each time points (n = 5). Nine frogs were used for compound action potential measurement. All experiments were examined and approved by the Committee of Animal Care of the West China Hospital Sichuan University (Ethical approval number, 2015014A). The practices of experiment were depending on the Guidance on the Care and Use of Laboratory Animals (U.S. National Institutes of Health Publication Publications No.80-23, revised in 1996). All procedures were strictly executed depending on the standard practice for biosecurity and institutional safety published by West China Hospital Sichuan University.

### Sciatic Nerve Block Model and Samples Collection

In brief, rats were anesthetized by inhaling 1%–2% isoflurane mixed with oxygen through an inhouse-made mask. Then, the rats were placed at right lateral position and inserted vertically using a 23-gauge injector at the middle of the line from the greater trochanter to ischial tuberosity (Binshtok et al., 2007; Zhang et al., 2017b). Once the bone has been contacted, 200 μl drug was injected.

According to the China Food and Drug Administration guidance for preclinical pharmacokinetic study, there should be more than three sampling points adjacent to the time to reach the maximum concentration. In our preliminary experiment, the time to maximum concentration of Levo-Bupi, as a single drug in the rat sciatic nerve block, was 0.5 h. Thus, to obtain an accurate value of the maximum concentration of Levo-Bupi, we added another time point at 0.33 h. Therefore, 200 μl blood sample was harvested *via* caudal vein from 10 rats after administration (half males and half females) at time points for QX-OH and LL-1: 0, 0.16, 0.5, 0.67, 1, 2, 3, 4, 5, 6, 8, 9, and 10 h; for Levo-Bupi: 0, 0.16, 0.33, 0.5, 0.67, 1, 2, 3, 4, 5, 6, 8, 9, and 10 h. Blood samples were maintained in heparinized polypropylene tube separately and then centrifuged at 877 × g for 10 min to gain plasma within 1 h. All the samples were kept at -80°C.

On the basis of previous pharmacodynamic studies, nine sampling points were used for determining drug concentration in muscle and sciatic nerve for QX-OH and LL-1: 0.5, 1, 2, 3, 4, 5, 6, 7, and 8 h; four sampling points for Levo-Bupi: 0.5, 1, 2, and 3 h. Due to the destructive sampling, five male rats were sacrificed at each time point for collecting muscle and sciatic nerve nearby the injection site. Then all samples were weighed and frozen at -80°C.

### Sample Preparation and Liquid Chromatography-Mass Spectrometry (LC-MS/MS) Analysis

According to previous study (Zhang et al., 2017a), plasma samples were deproteinized by acetonitrile solution with ropivacaine hydrochloride (internal standard, IS). The mixture was centrifuged for 10 min at 28,621 × g in 4°C after fully vortexed. The supernatant was diluted with ultrapure water (50:50, *v/v*). Muscle and sciatic nerve samples were homogenized by gentleMACS™ Dissociator (Miltenyi Biotec, Germany) and tissue grinder (model SCIENTZ-48, Ningbo, China), respectively. Tissue homogenate was mixed with IS solution by vortex and then centrifuged for 10 min at 28,621 × g. Then, the supernatant was diluted with ultrapure water at equal volume ratio. LC-MS/MS analysis system consist of Agilent 6460 triple quadrupole mass spectrometer and electrospray ionization source (Agilent Technologies, CA, USA). Chromatographic separation was adopted by Agilent Extend C18 column (100 mm × 3 mm, 3.5 μm) at 35°C with isocratic elution containing 0.05% formic acid and acetonitrile at the volume ratio of 78:22 at a flow rate of 0.3 ml/min. Mass spectrometry conditions were in a positive ionization mode: the sheath gas flow rate, 11.0 L/min; sheath gas heater temperature, 300°C; nebulizer pressure, 45 psi; capillary voltage, 3,500 V. MassHunter software was used to analyze data (B.04.00 Build 4.0.479.0, Agilent Technologies, USA).

### Pharmacokinetic Analysis

The parameters of pharmacokinetics were analyzed by DAS^®^ (Drug and Statistics, version 3.2.1, China) using noncompartmental model. In this study, the following pharmacokinetic parameters were calculated: the value of maximum concentration (C_max_) and the time to acquire (T_max_); the elimination half-life (t_1/2_); area under curve (AUC) from zero to the last time point (AUC_0–last_) and from zero to infinity (AUC_0–∞_); clearance (CL/F), and volume of distribution (V_d_/F).

### Record the Compound Action Potentials From Frog Sciatic Nerve Fibers

According to previous published researches (Matsushita et al., 2013; Ohtsubo et al., 2015). Nine of frogs were decapitated and then pithed. Then, the sciatic nerve was dissected from the lumbar plexus to the knee. The two ends of isolated sciatic nerves were carefully tied to the wires by threads and placed in Ringer solution (NaCl, 115.5 mmol/L; KCl, 2.0 mmol/L; CaCl_2_, 1.8 mmol/L; Na_2_HPO_4_, 1.3 mmol/L and NaH_2_PO_4_, 0.7 mmol/L; pH = 7.4).

The compound action potentials (CAPs) from frog sciatic nerve fibers was recorded by the biological function experiment system (BL-420F, Chengdu Techman Software, China). All practices were carried out at room temperature. The sciatic nerve was loosely placed in three platinum wires and then CAPs were recorded in air using a preamplifier. In this system, two of the platinum wires were used to record CAPs, and the other one was for stimulation. The parameters of stimulation were set as follows: intensity, 3 V; frequency, 1 Hz; rectangular pulses, 0.1 ms. The procedure should be performed quickly in order to avoid the nerve dry. The time of measurement should be less than 2 min. After CAPs examined, the sciatic nerve was put back into Ringer solution with drugs. In this study, we compared the amplitude of CAPs after using drugs with baseline to present the degree of inhibition from frog sciatic nerve, and which was analyzed using the following equation: CAPs amplitude (%) = CAPs _measurement_/CAPs _baseline_ × 100%.

### Cellular Drug Uptake Assay

HT22 cell line, a sub-line derived from HT4 cells that were originally immortalized from primary mouse hippocampal neuron, were a generous gift from Dr. Huang (West China Second University Hospital, Sichuan University, China). HT22 cells were cultured in DMEM with high glucose (HyClone, Utah, USA) supplemented with 10% fetal bovine serum (Gibco, New York, USA) and 1% penicillin and streptomycin (HyClone, Utah, USA) in a humidified incubator with 5% CO_2_ and 95% air at 37°C.

According to previous study (Brenneis et al., 2014), cells were plated on 35 mm 6-well dishes (2.5 × 10^5^ per well). When cells reached confluence at 70%–80%, the medium was removed, and plates washed with PBS (NaCl, 137.0 mmol/L; KCl, 2.7 mmol/L; KH_2_PO_4_, 1.5 mmol/L and Na_2_HPO_4_·12H_2_O, 8.0 mmol/L; pH = 7.4). Thirty wells were divided to incubate with LL-1, 35 mM QX-OH and 10 mM Levo-Bupi for 10 min, and then washed four times with PBS. Cells were translated to polypropylene tube. The cell suspensions were centrifuged at 72 × g and the supernatants were removed by pipette. All samples were frozen at -80°C before testing. Samples were homogenized with 100 µL of saline using a tissue grinder. After mixing 50 μl homogenate, 50 μl acetonitrile, and 10 μl IS, then the mixture was centrifuged for 10 min at 28,621 × g, and the supernatant was injected for analysis.

### Statistical Analysis

All statistical analyses were calculated through SPSS (version 23, SPSS, Chicago, IL, USA). All data should be examined by tests for standard normal distribution and homogeneity of variance. If the data are without normal distribution or equal variance, it should be analyzed by the method of nonparametric tests. For the CAPs amplitude, it used two-way repeated measures analysis of variance to compare the difference of drug and time, and then followed with the method of least significant difference (LSD) for pairwise comparison to compare the difference among drugs in the same time point. For the half-time and time to block CAPs, it was conducted by Kruskal–Wallis test due to its unequal variance, and the Cmax, Tmax and the cellular drug uptake assay were statistically analyzed using independent-sample T test. The standard of statistical significance was set at p < 0.05.

## Results

### Pharmacokinetics of LL-1

**Figure 1:**
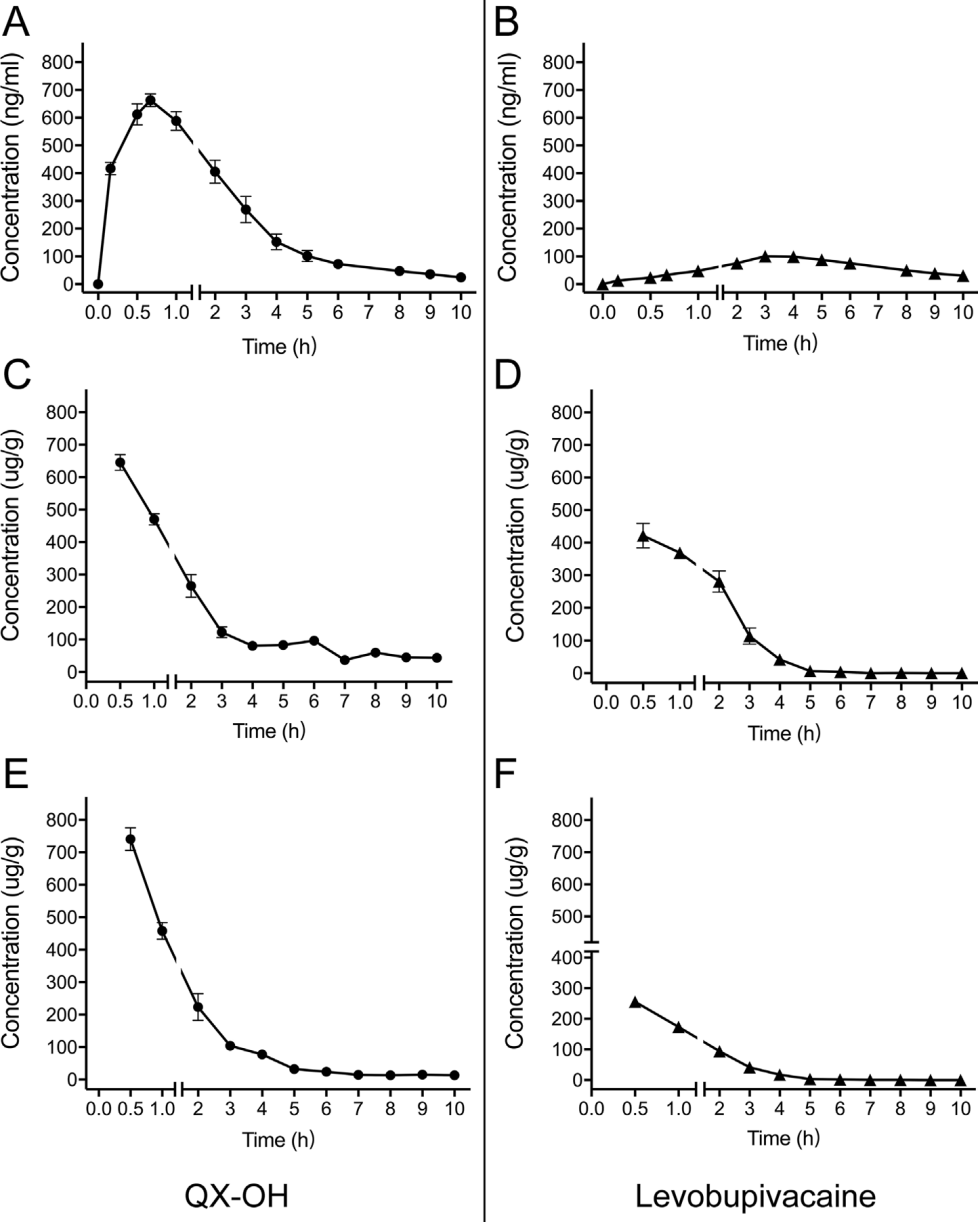
Mean concentration-time profiles of LL-1 in plasma **(A–B)**, sciatic nerve (**C**–**D**) and muscle **(E–F)** after sciatic nerve block in rats. (n = 10 for plasma, and n = 5 per time point for muscle and sciatic nerve). Data are expressed as mean ± SEM. Levo-Bupi, levobupivacaine; LL-1, 35 mM QX-OH/10 mM Levo-Bupi.

After sciatic nerve block, the LL-1 concentration-time profiles were determined in the plasma, sciatic nerve, and muscle. The data are presented in **Figure 1**, and the calculated pharmacokinetic parameters for LL-1 are shown in **Table 1**. The C_max_ values of QX-OH and Levo-Bupi were 727.22 ± 43.38 and 256.02 ± 28.52 μg/g in muscle, 634.26 ± 36.04 and 429.63 ± 48.64 μg/g in sciatic nerve, and 711.71 ± 25.14 and 114.40 ± 10.19 ng/ml in plasma, respectively. The C_max_ of QX-OH in sciatic nerve did not significantly differ from that in muscle (p = 0.058). In contrast, the C_max_ of Levo-Bupi was higher in sciatic nerve (429.63 ± 48.64 μg/g) than that in muscle (256.02 ± 28.52 μg/g, p = 0.008). In LL-1, the absorption of QX-OH into circulation (0.71 ± 0.06 h) was more rapid than that of Levo-Bupi (4.11 ± 0.39 h, p = 0.003). The half-time of QX-OH in plasma and local tissues had no significant difference (p = 0.329), with the values of 2.64, 3.20, and 3.79 h in plasma, muscle, and sciatic nerve, respectively. The elimination profile of Levo-Bupi differed from that of QX-OH, which was slower eliminated from plasma (4.89 ± 1.77 h, p = 0.036) than from muscle (1.38 ± 0.60 h) or sciatic nerve (1.28 ± 0.74 h). The computed AUC values of QX-OH from zero to the last time point were 1,487.72 ± 82.20 μg*h/g in sciatic nerve, 1,188.29 ± 62.38 μg*h/g in muscle, and 1,913.72 ± 148.7 ng*h/ml in plasma. The AUC values of Levo-Bupi from zero to the last time point were 935.82 ± 74.35 μg*h/g in sciatic nerve, 417.58 ± 43.46 μg*h/g in muscle, and 651.23 ± 47.39 ng*h/ml in plasma.

**Table 1:** Tissue and systemic pharmacokinetic parameters of LL-1 after sciatic nerve block in rats (Mean ± SEM).

	Plasma^a^		Muscle^b^		Sciatic nerve^b^	
	QX-OH	Levobupivacaine	QX-OH	Levobupivacaine	QX-OH	Levobupivacaine
C_max_	711.71 ± 25.14	114.40 ± 10.19	727.22 ± 43.38	256.02 ± 28.52	634.26 ± 36.04	429.63 ± 48.64
T_max_ (h)	0.71 ± 0.06	4.11 ± 0.39	0.5	0.5	0.5	0.5
t_1/2_ (h)	2.64 ± 0.43	4.89 ± 1.77	3.20 ± 1.46	1.38 ± 0.60	3.79 ± 1.07	1.28 ± 0.74
V_d_/F	4.41 ± 0.46	3.81 ± 0.70	9.22 ± 4.07	3.01 ± 1.21	7.81 ± 1.38	1.23 ± 0.80
CL/F	1.27 ± 0.12	0.78 ± 0.10	2.02 ± 0.10	1.59 ± 0.17	1.50 ± 0.14	0.70 ± 0.05
AUC_0–t_	1,913.72 ± 148.7	651.23 ± 47.39	1,188.29 ± 62.38	417.58 ± 43.46	1,487.72 ± 82.20	935.82 ± 74.35
AUC_0–∞_	1,977.78 ± 158.7	819.30 ± 78.85	1,251.11 ± 62.72	417.86 ± 43.49	1,715.06 ± 189.94	935.88 ± 74.39

CL/F apparent clearance, V_d_/F apparent volume of distribution, AUC stand for area under curve.

a = 10. Unit for plasma are ng/ml, L, L/h, and ng*h/ml for C_max_, V_d_/F, CL/F, and AUC, respectively.

b = 5 per time point. Unit for muscle and sciatic nerve are µg/g, L, L/h, and µg*h/g for C_max_, V_d_/F, CL/F, and AUC, respectively.

### Pharmacokinetics of Levobupivacaine

**Figure 2:**
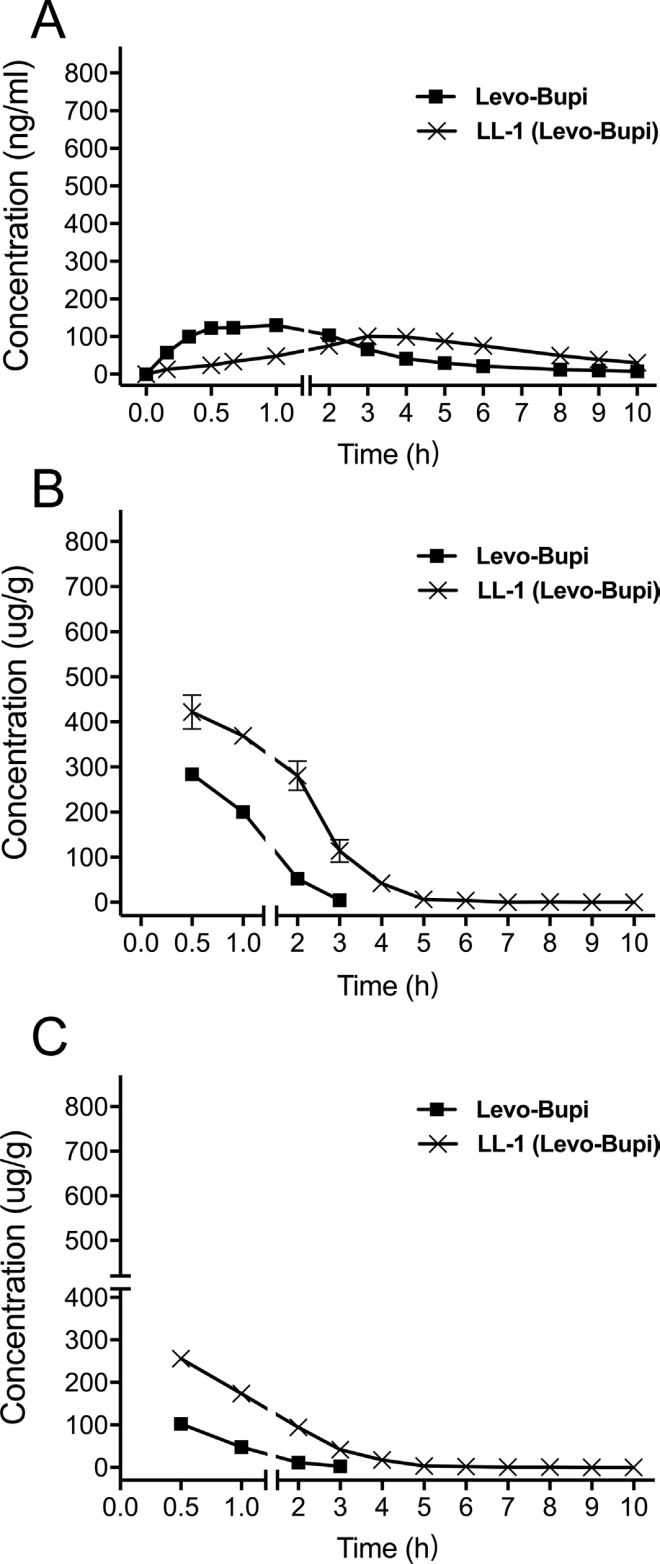
Mean concentration-time profiles of Levobupivacaine in plasma **(A)**, sciatic nerve **(B)**, and muscle **(C)** after sciatic nerve block in rats. (n = 10 for plasma, and n = 5 per time point for muscle and sciatic nerve). Data are expressed as mean ± SEM. Levo-Bupi, levobupivacaine; LL-1, 35 mM QX-OH/10 mM Levo-Bupi.

In a previous study (Yu et al., 2002), the C_max_ of bupivacaine was reached at 0.5 h after subcutaneous administration. However, the T_max_ of Levo-Bupi in LL-1 was 4.11 ± 0.39 h. Therefore, we tested Levo-Bupi as a single drug in the rat sciatic nerve block model and determined its plasma concentration. The concentration-time profiles of Levo-Bupi are presented in **Figure 2**, and the calculated pharmacokinetic parameters are shown in **Table 2**. When Levo-Bupi was used alone, the T_max_ in plasma was 1.07 ± 0.16 h (**Figure 2A**). Interestingly, the T_max_ of Levo-Bupi in the plasma was increased by four times in combination with QX-OH (4.11 ± 0.39 h, p < 0.001). The C_max_ of Levo-Bupi, used alone, (142.45 ± 13.04 ng/ml) did not significantly differ from that in LL-1 (p = 0.114). In contrast, Levo-Bupi, used alone, had a faster elimination (CL = 1.32 ± 0.08 L/h, p = 0.001; t_1/2_ = 2.61 ± 0.31 h, p = 0.199). In sciatic nerve block model, the drugs were administrated adjacent to sciatic nerve and then absorbed into circulation. Based on this assumption, we predicted that the absorption delay of Levo-Bupi was related to its accumulation in local tissues. So, we further measured the drug concentration in sciatic nerve and adjacent muscle. When Levo-Bupi was used as a single drug, its concentration in sciatic nerve went from 284.01 ± 32.25 to 4.64 ± 3.66 μg/g; in LL-1, the Levo-Bupi concentration was increased at 1.5 to 24 times in LL-1 at the same time points (422.06 ± 83.62 to 113.63 ± 55.91 μg/g, **Figure 2B**). Similarly, in the single-drug test, the Levo-Bupi concentration in muscle went from 102.25 ± 15.28 to 2.84 ± 3.25 μg/g, but was increased by 2 to 15 times in the LL-1 at the same time points (256.02 ± 42.78 to 41.42 ± 20.69 μg/g, **Figure 2C**). These results showed that QX-OH could promote Levo-Bupi accumulation in local tissues, which could delay its absorption into blood circulation.

**Table 2:** The pharmacokinetic parameters of Levobupivacaine in plasma after sciatic nerve block in rats (Mean ± SEM).

	Levobupivacaine alone	Levobupivacaine in LL-1
C_max_ (ng/ml)	142.45 ± 13.04	114.40 ± 10.19
T_max_ (h)	1.07 ± 0.16	4.11 ± 0.39
t_1/2_ (h)	2.61 ± 0.31	4.89 ± 1.77
V_d_/F (L)	4.81 ± 0.50	3.81 ± 0.70
CL/F (L/h)	1.32 ± 0.08	0.78 ± 0.10
AUC_0–t_ (ng*h/ml)	471.25 ± 23.28	651.23 ± 47.39
AUC_0–∞_ (ng*h/ml)	508.69 ± 31.47	819.30 ± 78.85

CL/F apparent clearance, V_d_/F apparent volume of distribution, AUC stand for area under curve.

### Effect of Drugs on the CAPs of the Frog Sciatic Nerve

**Figure 3:**
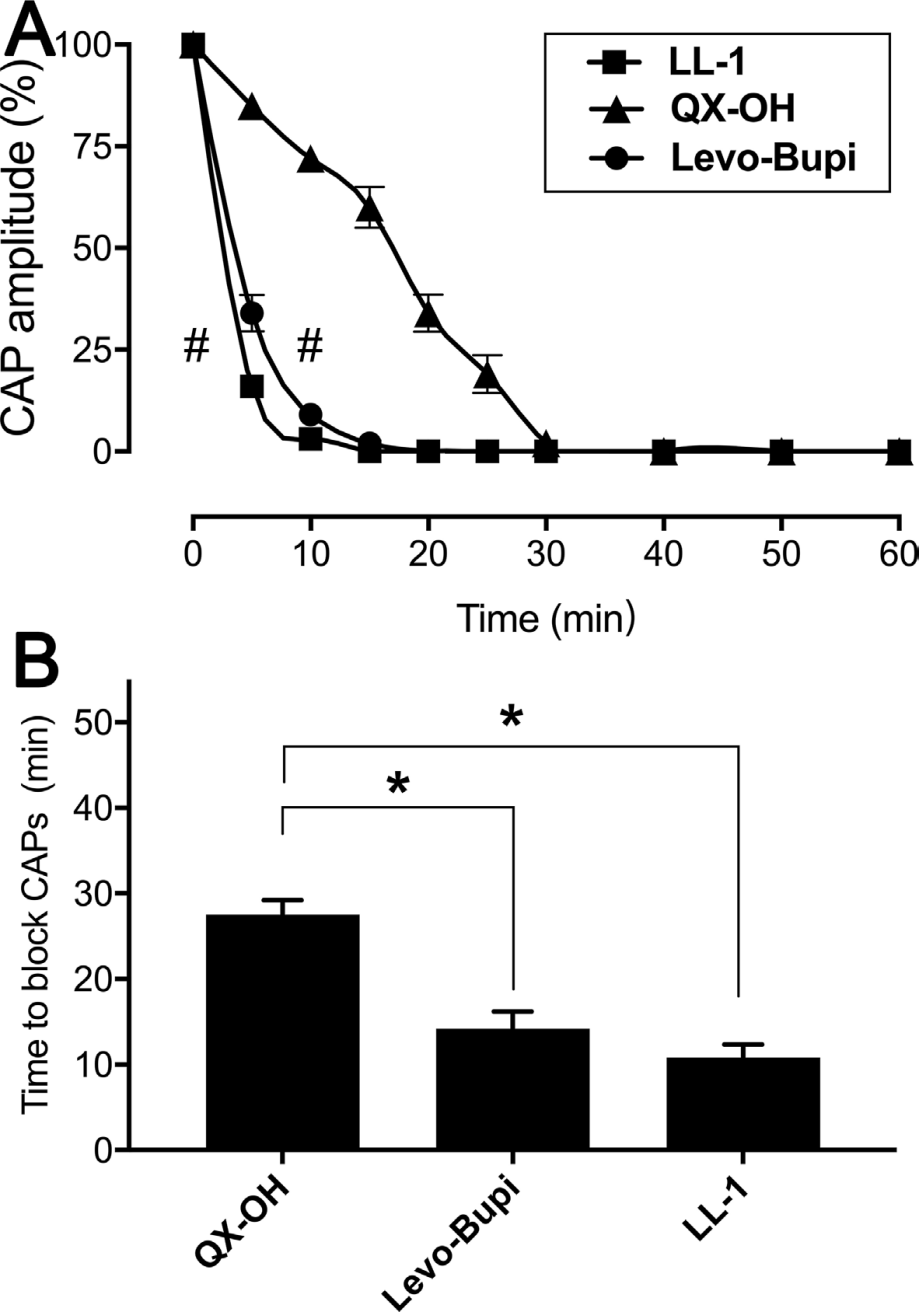
Effects of LL-1, QX-OH and Levobupivacaine on frog sciatic nerve CAPs. **(A)** the CAP amplitude; **(B)** The time to block CAPs, n = 6 in each group). Data are expressed as mean ± SEM. * p < 0.05, # p < 0.001. Levo-Bupi, levobupivacaine, LL-1: 35 mM QX-OH/10 mM Levo-Bupi.

Local anesthetics are known to prevent the action potentials of nerve blocking the sensory and motor function (Miller et al., 2009). In local anesthesia animal models, administration of LL-1 was associated with longer duration and shorter onset time than administering the single drugs, QX-OH and Levo-Bupi, at the same concentration (Yin et al., 2019). Monitoring the CAPs in frog sciatic nerve fibers, we confirmed the effect of LL-1, 35 mM QX-OH, and 10 mM Levo-Bupi. The amplitude of CAPs was decreased with the exposure time (**Figure 3A**). Compared with QX-OH, the CAPs were affected significantly by LL-1 (p < 0.001) and Levo-Bupi (p < 0.001). Within the first 5 min, the CAPs amplitude was reduced to 16%, 34% and 85% by LL-1, Levo-Bupi and QX-OH, respectively. The soak time required to block CAPs was longer with QX-OH (27.50 ± 1.71 min, **Figure 3B**) than with LL-1 (10.83 ± 1.53 min, p = 0.003) or Levo-Bupi (14.17 ± 2.01 min, p = 0.034).

### Cellular Drug Uptake Assay

**Figure 4:**
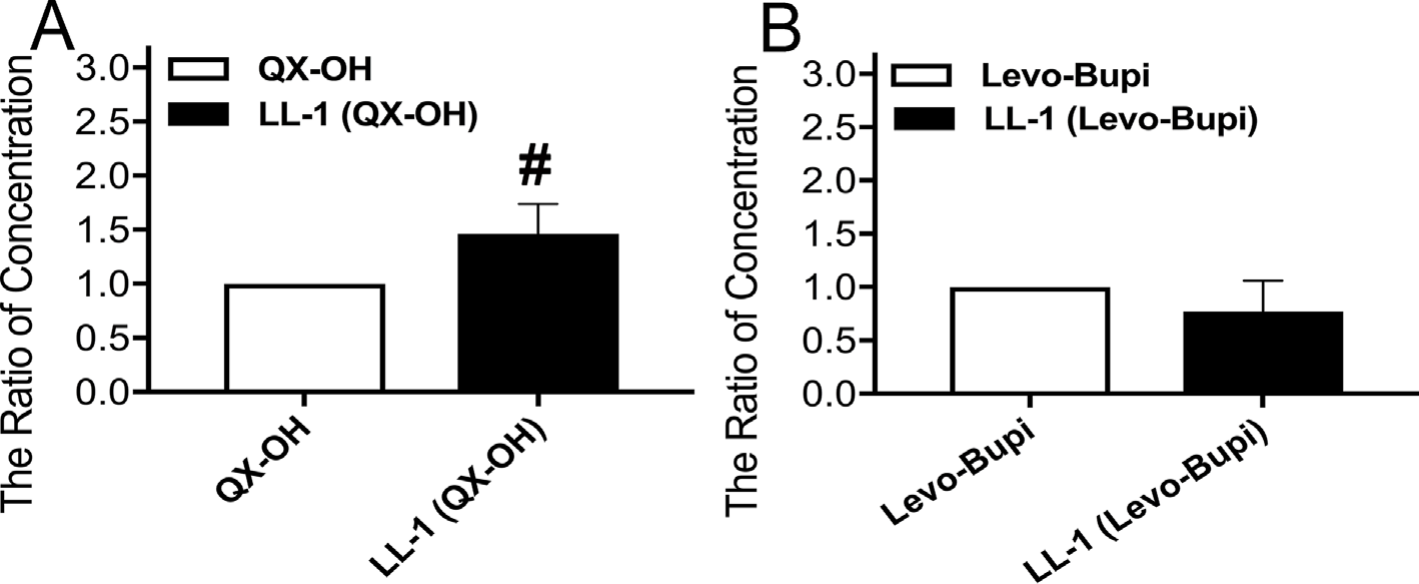
The ratio of concentration by cellular drug uptake in HT22 cells (n = 10 in each group). **(A)** QX-OH; **(B)** Levo-Bupi. Data are expressed as mean ± SEM. # p < 0.001. Levo-Bupi, levobupivacaine; LL-1, 35 mM QX-OH/10 mM Levo-Bupi.

The extended duration in pharmacodynamic study cannot be easily explained only by the slow absorption of Levo-Bupi. Local anesthetics block the action potentials by penetrating into cell and inhibiting the voltage-gated sodium channels (Narahashi et al., 1970; Fink, 1989; Butterworth and Strichartz, 1990). Hence, we tested whether Levo-Bupi can promote cellular QX-OH uptake. Levo-Bupi accelerated membrane permeation of QX-OH and its accumulation in the cells (**Figure 4A**, p < 0.001). However, the cellular uptake of Levo-Bupi had no difference (**Figure 4B**, p = 0.067).

## Discussion

In this study, we performed the preclinical pharmacokinetic analysis of a novel long-acting local anesthetic, a fixed-dose combination of QX-OH/Levo-Bupi, in rat plasma, muscle, and sciatic nerve. We further explored the underlying mechanism of the extended duration of local anesthetic effects produced by the combination. The pharmacokinetic studies were performed in the sciatic nerve block model.

Previous reports indicated that LL-1 had a longer duration with a rapid onset (Zhao et al., 2017a; Yin et al., 2019). The duration of one drug is closely related to its pharmacokinetic feature. Many factors can affect the pharmacokinetics of a local anesthetic, such as the molecular structure, physicochemical properties, method of administration, local blood flow, and drug–drug interaction. In this study, we proved that QX-OH delayed the absorption of Levo-Bupi from local tissues into circulation. Meanwhile, Levo-Bupi accelerated QX-OH to accumulate into cells. There are two possible reasons for the delayed absorption of Levo-Bupi, decreased local blood flow or drug–drug interactions. In clinical practice, epinephrine, a vasoconstrictor agent, is used to increase the duration of local anesthesia. In the inferior alveolar nerve block (Keesling and Hinds, 1963; Dagher et al., 1997), the T_max_ of Levo-Bupi was doubled by combining it with epinephrine in the transversus abdominis plane block (Corvetto et al., 2012). Thus, epinephrine extended the T_max_ of local anesthetics by decreasing local blood flow. The T_max_ of Levo-Bupi in the plasma was increased by four times after combine with QX-OH. However, the underlying mechanism of the effect of QX-OH is not fully understood. We considered that this effect might have been generated by a decreased local blood flow. However, in a recent study, Jang, et al. mixed lidocaine with trivalent PO_4_^3-^ to generate a lidocaine/multivalent ion complex by ionic interaction, which promoted a prolonged period of lidocaine release and a long duration of nerve blockade (Jang et al., 2017). Meanwhile, Yin, et al. investigated the effect of differently charged liposomes on the duration of QX-314. Anionic liposomes produced a longer duration of the nerve blockage when compared with the cationic and neutral liposomes. Interestingly, QX-314 was attached to the surface of anionic liposomes by electrostatic interactions (Yin et al., 2017). Similar to the results with QX-OH, the T_max_ of Levo-Bupi with in combination with QX-314 was extended to 4 h in the sciatic nerve block (**Supplementary Figure 1A**). However, lidocaine did not have a similar effect on the absorption of Levo-Bupi (**Supplementary Figure 1B**). In solution, traditional local anesthetics, such as lidocaine and bupivacaine, are in a rapid equilibrium between the uncharged and charged status. Based on the chemical structures, the most important difference between the traditional local anesthetics and QX-OH and QX-314 is that the latter two compounds are permanently charged in solution. Meanwhile, Levo-Bupi owns an ione electron pair in the nitrogen atom of tertiary ammonium group. Thus, we considered whether the underlying mechanism of QX-OH-induced the delayed absorption of Levo-Bupi is ionic interactions.

In the Results section, we demonstrated that Levo-Bupi accelerated the cellular accumulation of QX-OH, which was an important mechanism to extend the duration of LL-1. In previous study, bupivacaine enhanced the cellular uptake of QX-314 in RAW, N1E and CHO cells (Brenneis et al., 2014). In addition, bupivacaine, a transient receptor potential (TRP) channels agonist, promote QX-314 to permeate the membrane *via* TRP channels (Meyers et al., 2003; Binshtok et al., 2007; Chung et al., 2008; Banke and Wickenden, 2009; Chen et al., 2009; Kim et al., 2010; Rivera-Acevedo et al., 2011; Stueber et al., 2016). Based on an analogical molecular structure with QX-314, we considered that QX-OH might be accumulated into cellular through TRP channels as QX-314 which was assist of Levo-Bupi as a TRP channels agonist.

## Conclusion

The preclinical pharmacokinetic study of a novel long-acting local anesthetic, a fixed-dose combination of QX-OH/Levo-Bupi, in rat plasma and local tissues was accomplished. Then, the prolonged nerve blockade may be related to the effect of interaction between QX-OH and Levo-Bupi. QX-OH delayed the absorption of Levo-Bupi from the local tissue into circulation, and Levo-Bupi accelerated QX-OH to accumulate into cells.

## Data Availability

The raw data supporting the conclusions of this manuscript will be made available by the authors, without undue reservation, to any qualified researcher.

## Author Contributions

YZ, QY, JL, and WZ conceived and designed the project. YZ, QY, DG, YK, and JY performed the experiments. YZ and WZ analyzed the data. YZ wrote the manuscript. JL and WZ revised the manuscript.

## Funding

This work was supported by the Post-Doctor Research Project, West China Hospital, Sichuan University (2019HXBH012) and the National Science and Technology Major Project, Ministry of Science and Technology of the People’s Republic of China (No. 2014ZX09101001-003).

## Conflict of Interest Statement

The authors declare that the research was conducted in the absence of any commercial or financial relationships that could be construed as a potential conflict of interest.

## Supplementary Material

Figure S1 Drug-time curve of QX-314/Levo-Bupi (35 mM/10 mM, A) and Lidocaine/Levo-Bupi (35 mM/10 mM, B) in plasma after sciatic nerve block in rats. (n = 10 in each group). Data are expressed as mean ± SEM. Levo-Bupi: levobupivacaine
