# Supplementary figures and images for: The Preclinical Pharmacological Study of a Novel Long-Acting Local Anesthetic, a Fixed-Dose Combination of QX-OH/Levobupivacaine, in Rats
Source: Front Pharmacol. 2019 Aug 15;10:895. doi: 10.3389/fphar.2019.00895 (PMC6704344; doi:10.3389/fphar.2019.00895)

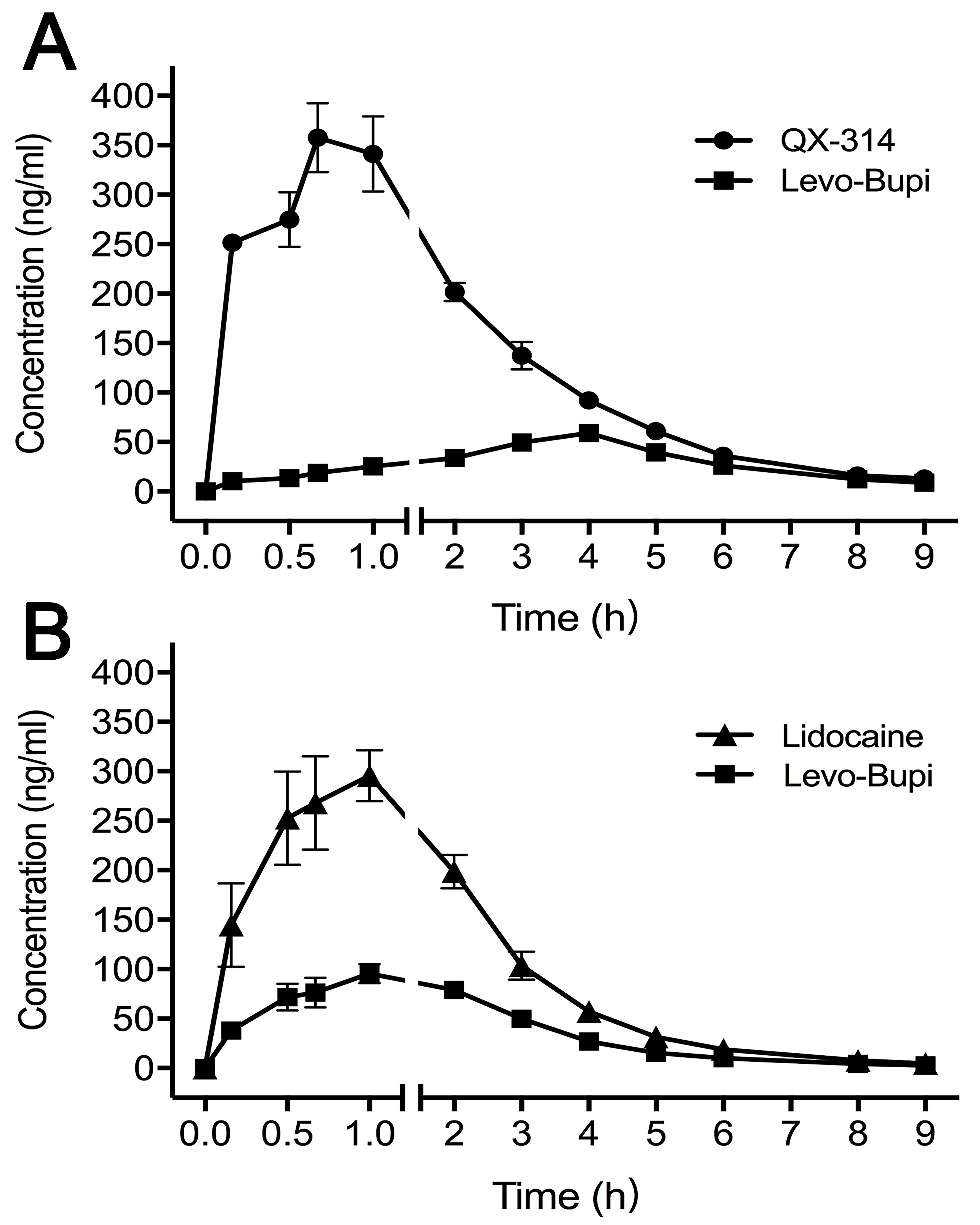

Supplement: Figure S1 — Drug-time curve of QX-314/Levo-Bupi (35 mM/10 mM, A) and Lidocaine/Levo-Bupi (35 mM/10 mM, B) in plasma after sciatic nerve block in rats. (n = 10 in each group). Data are expressed as mean ± SEM. Levo-Bupi: levobupivacaine [file Image_1.tif]
